# Influence of maternal body mass index on gestational weight gain and birth weight: A comparison of parity

**DOI:** 10.3892/etm.2013.1167

**Published:** 2013-06-19

**Authors:** TAKAKO CHIBA, SATOKO EBINA, IKUO KASHIWAKURA

**Affiliations:** 1 Departments of Radiological Life Sciences and; 2 Disability and Health, Hirosaki University Graduate School of Health Sciences, Hirosaki, Aomori 036-8564, Japan

**Keywords:** maternal physique, gestational weight gain, body mass index, birth weight

## Abstract

Previous studies have revealed correlations among prepregnancy body mass index (BMI), gestational weight gain and the birth weight of the infant. However, as a variety of indices relating to the physique have been used to assess the optimal weight of pregnant women, no conclusions have yet been established regarding the Japanese population. Therefore, the aim of this study was to analyze the correlations among prepregnancy BMI, gestational weight gain and the birth weight of the infant in primiparous and multiparous females. The study was a retrospective analysis of pregnancy charts from a single birthing center from August 1998 to the end of September 2007. The subjects were primiparous (n=220) and multiparous (n=340) females, and the mean prepregnancy weights of the two groups were 52.8±8.8 and 54.3±9.0 kg, respectively. The mean prepregnancy BMI of the primiparous females was 20.8±3.1 kg/m^2^, compared with 21.6±3.5 kg/m^2^ for the multiparous females, and the mean birth weights of the infants were 3,153.0±364.1 g and 3,262.3±370.4 g for primiparous and multiparous females, respectively. When the correlation between the maternal factors and the birth weight of the infant was analyzed, the birth weight was revealed to be positively correlated with delivery weight and gestational weight gain in primiparous females. However, no correlations were observed between the birth weight of the infant and prepregnancy weight or BMI. In multiparous females, birth weight was revealed to be positively correlated with prepregnancy weight, BMI and the maternal delivery weight; however, no correlation was observed between the birth weight of the infant and gestational weight gain. The results of the present study also demonstrated that there were significant differences between the primiparous and multiparous females, with regard to gestational weight gain and weight reduction following delivery. The study indicated that the factors influencing birth weight may be different for primiparous and multiparous females.

## Introduction

In general, the female body undergoes marked changes during pregnancy, with alterations in nutritional status, metabolism, endocrinology and circulation. Gestational weight gain occurs as a result of the growth of the fetus and the mother: The fetal weight increases and fetal appendages develop, and there is an increase in the size of the mother’s uterus, breasts, circulating blood volume and extracellular fluid volume (1). Hytten and Leitch have estimated through metabolic analysis that the physiological weight gain in pregnant females with an average build is ∼12.5 kg (2). By contrast, the average weight gain observed in the average Japanese female during pregnancy is only 9.8–10.5 kg (3). At present, it is accepted that there is a positive correlation between gestational weight gain and the birth weight of the the infant (4). However, this correlation appears to decrease when the prepregnancy body mass index (BMI) of the female is high (4). A study performed in the USA has demonstrated that a higher weight gain in females during pregnancy resulted in a large baby and an increased frequency of cesarean section, with the birth of low-weight infants a rarity (5). In Japan, the correlation between gestational weight gain and pregnancy-induced hypertension (PIH) is particularly prominent, and priority has been allocated to helping pregnant females limit their weight gain. This is one reason for the recent reduction in infant birth weights in Japan. In addition, it is possible that the desire in young females to be thin, thereby reducing their BMI, is a contributory factor in birth weight reduction.

Maternal and fetal outcomes are at risk with raised and lowered prepregnancy BMIs. Females with a prepregnancy BMI <18.5 kg/m^2^ are at risk of premature delivery and a low birth weight of the infant (6–9). However, studies have revealed that females with a BMI >29 kg/m^2^ are at risk of PIH, gestational diabetes, cesarean section, a large baby and neural tube defects (10–14). Similar to prepregnancy BMI, gestational weight gain also has a significant impact on maternal and fetal outcomes. Gluckman and Hanson previously reiterated the concept of the developmental origins of health and disease (DOHaD), stating that ‘environmental factors acting during the phase of developmental plasticity interact with genotypic variation to change the capacity of the organism to cope with its environment later in life’ (15). The maintenance of appropriate nutrition in prepregnant and pregnant females is important in the prevention of future illness.

Previous studies have revealed correlations among prepregnancy BMI, gestational weight gain and resultant infant birth weight. However, as a variety of indices relating to the physique have been used to assess the optimal weight of pregnant women, no conclusions have yet been established regarding the Japanese population. Therefore, the aim of this study was to investigate the correlations among prepregnancy BMI, gestational weight gain and the birth weight of the infant in primiparous and multiparous females. The study was a retrospective analysis of the deliveries at a single birthing center in a provincial city in Japan over a 10-year period.

## Materials and methods

### Study design

This study was a retrospective analysis of pregnancy charts from a single birthing center (the Fukushi Birth Center, Goshogawara, Aomori, Japan) from August 1998 until the end of September 2007. The study was conducted in compliance with the principles of the Declaration of Helsinki (as revised in Seoul, 2008) and the ethical guidelines for epidemiological research provided by the Ministry of Education, Culture, Sports, Science and Technology as well as the Ministry of Health, Labour and Welfare in Japan (2008). All data used in the present study were coded and obtained from the pregnancy charts of subjects without disclosing their identity.

### Selection of the study population

The criteria for inclusion were a singleton, low-risk, full-term pregnancy (duration, 37–42 weeks), resulting in a spontaneous vaginal delivery. Mothers with chronic diseases (including diabetes, hypertension and hyperthyroidism), gestational diabetes and PIH were excluded from this study. In accordance with the World Health Organization (16) and the Japan Society for the Study of Obesity (17), prepregnancy BMI was classified into four groups: underweight (<18.5 kg/m^2^), normal (18.5 to <25 kg/m^2^), overweight (25 to <30 kg/m^2^) and obese (≥30 kg/m^2^).

### Data collection

A chart review was conducted of the pregnancy charts of 579 females who delivered at the Fukushi Birth Center. Records with unknown or missing data regarding obstetric factors were not included. A total of 560 cases were available for the final analysis. Perinatal data was collected on maternal age, parity, self-reported prepregnancy weight, prepregnancy BMI, gestational weight gain, chronic diseases, delivery mode, duration of pregnancy, duration of labor, neonatal gender, neonatal size and the weight of the mother, one month postpartum.

### Data analysis

Statistical analysis was performed using SPSS software, version 16.0 (SPSS Japan, Inc., Tokyo, Japan) for Windows. Descriptive statistics are presented as the arithmetic mean ± standard deviation. A two-sample t-test was performed to determine differences across the three groups, while a χ^2^ analysis was used to analyze categorical variables. Univariate analysis was performed using the Pearson’s correlation coefficient, and a multiple linear regression analysis was performed to determine any correlation between the birth weight of the infant (object functions) and maternal factors (explanatory variables). P<0.05 was considered to indicate a statistically significant difference.

## Results

### Demographics and characteristics of the study population

The population demographics and characteristics are presented in Table I. The subjects were either primiparous (n=220) or multiparous (n=340) females, with an age range from 17 to 41 years (mean age, 24.1±3.8 and 28.2±4.3 years, respectively). The mean prepregnancy weight was 52.8±8.8 kg (range, 38–100 kg) in primiparous and 54.3±9.0 kg (range, 40–96 kg) in multiparous females, while the mean prepregnancy BMI was 20.8±3.1 kg/m^2^ (range, 15.8–35.4 kg/m^2^) in primiparous and 21.6±3.5 kg/m^2^ (range, 16.2–35.3 kg/m^2^) in multiparous females. The maternal age, prepregnancy weight and prepregnancy BMI were significantly higher in the multiparous than in the primiparous females (P<0.05). Among the primiparous females, the mean gestational weight gain was 12.7±4.3 kg (range, 1.8–25.5 kg), while among the multiparous females it was 11.4±4.3 kg (range, −2.0–28.0 kg). The mean postpartum weight loss was 7.6±2.3 kg (range, 0–14.5 kg) and 7.1±2.3 kg (range, 1.0–16.6 kg) in primiparous and multiparous females, respectively. The gestational weight gain and postpartum weight loss were significantly higher in primiparous than in multiparous females (P<0.05).

The overall male infant birth rate was 50.9% (Table II). The mean birth weights of the infants were 3,153.0±364.1 g (range, 2,160–4,220 g) and 3,262.3±370.4 g (range, 2,190–4,540 g) for the primiparous and multiparous females, respectively, while the overall rate of low birth weight was 1.4%. Infant birth weight, head circumference and chest circumference were all significantly higher in multiparous than in primiparous females (P<0.05). In addition, the low birth weight rate was significantly higher in primiparous than in multiparous females (P<0.05).

### Correlation between maternal factors and infant birth weight

**Figure 1.**
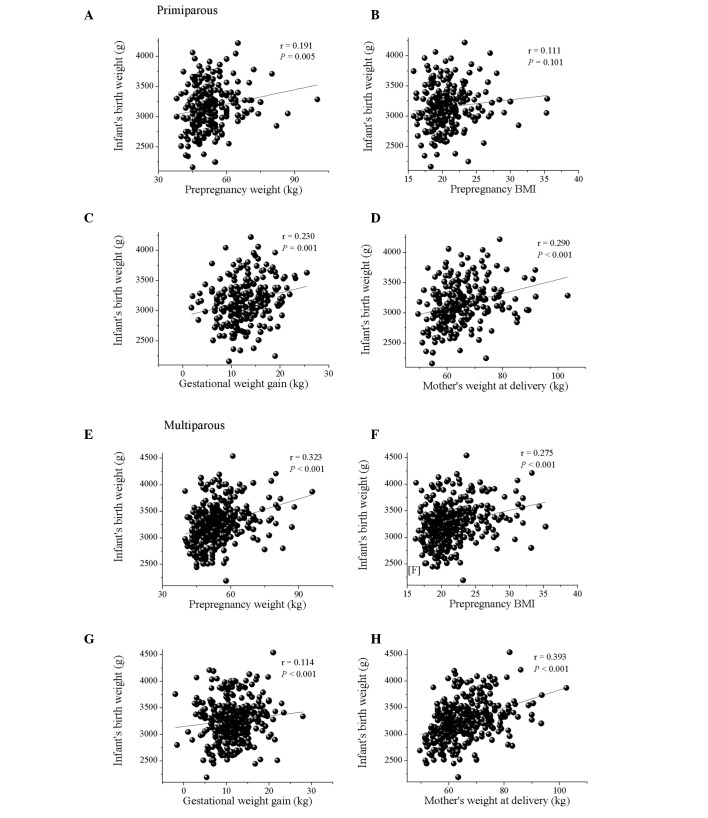
Correlations between maternal factors and birth weight among (A–D) primiparous (n=220) and (E–H) multiparous (n=340) females. The maternal factors are: (A and E) Prepregnancy weight; (B and F) prepregnancy body mass index (BMI; kg/m^2^); (C and G) gestational weight gain; and (D and H) delivery weight. Significantly positive correlations are observed in (C) gestational weight gain and (D) maternal delivery weight in the primiparous females, and in (E) prepregnancy weight, (F) prepregnancy BMI and (H) maternal delivery weight (Pearson’s correlation coefficient) in the multiparous females.

The analysis of the correlation between maternal factors and infant birth weight revealed that the birth weight was positively correlated with prepregnancy weight, prepregnancy BMI, delivery weight and the duration of the pregnancy. However, no correlations were observed among birth weight, maternal age and gestational weight gain (data not shown). In addition, birth weight in the nonsmoking group was significantly higher than that in the smoking group (3,234±372.0 versus 3,153.9±364.0 g, P<0.05, data not shown). When comparing parity with the maternal factors, infant birth weight was positively correlated with maternal delivery weight and gestational weight gain in primiparous females (Fig. 1). However, no correlations were observed among birth weight, prepregnancy weight and prepregnancy BMI. In multiparous females, the birth weight of the infant was positively correlated with prepregnancy weight, prepregnancy BMI and maternal delivery weight, but no correlation was observed among birth weight and gestational weight gain. Moreover, no significant differences were revealed between the smoking and nonsmoking groups in either primiparous or multiparous females (data not shown).

## Discussion

This study analyzed the correlation between prepregnancy BMI, gestational weight gain and infant birth weight in primiparous and multiparous females, and was performed using a retrospective analysis of pregnancy charts from a single birthing center from August 1998 until the end of September 2007. The mean age of the subjects was 26.6±4.6 years, which is lower than the mean value for Japanese females (18). The rate of females who were multiparous was also higher (60.7%) than the mean rate for Japan as a whole. The average birth weight of the infants was 3,219.3±371.5 g, indicating that the rate of low birth weight was 1.4%. This value revealed that there were fewer infants with a low birth weight compared with the average for Japanese females. One explanation may be that the birthing center only accepted females with normal pregnancies that were expected to result in a safe delivery and require little medical intervention. The average prepregnancy BMI was 21.3±3.3 kg/m^2^, and the underweight group (those with a BMI <18.5 kg/m^2^) accounted for 15.2% of the total subject population. There has been a sharp increase in the number of underweight females aged 20–29 years in Japan, and this study reflected that trend (19). This is therefore becoming a greater social issue.

It is accepted that there is a linear correlation between maternal prepregnancy BMI and the mean birth weight of the infant (20). In addition, various other factors have been observed to affect the birth weight of the infant, as follows: the gender of the infant, gestational age, smoking, obstetric history and genetic predisposition (21,22). Neonatal anthropometric charts for gestational age are widely used by obstetricians and pediatricians to predict neonatal risk and to monitor infants. According to the anthropometric charts used in Japan, certain differences have been revealed with regard to the gestational weight gain in primiparous and multiparous females from ∼32 weeks of gestation (23). In addition, a recent large-scale study observed that although there were differences in the timing of the appearance of weight differences, the birth weights of the infants of multiparous females were higher than those of primiparous females (24). Although the mechanism for these observed weight differences has yet to be elucidated, the birth weight of the infant was generally higher for multiparous than for primiparous females. The results of the present study concurred with this, with the infants born to multiparous females being heavier than those born to primiparous females (Table II). Furthermore, the gestational weight gain was observed to be weakly correlated with the birth weight of the infant in primiparous females (Fig. 1C). By contrast, prepregnancy weight and BMI were revealed to be positively correlated with birth weight in multiparous females (Fig. 1E and F). The relationship between maternal physique and gestational weight gain has been well established. However, this study suggested that the factors influencing infant birth weight were different for primiparous and multiparous females. In addition, the fact that prepregnancy weight and BMI were significantly higher in multiparous than in primiparous females may have influenced the differences in birth weight between the two groups.

The primiparous and multiparous females were also compared in terms of changes in maternal physique during pregnancy and at one month postpartum. Prepregnancy BMI was observed to be higher in multiparous than in primiparous females, and gestational weight gain and weight reduction following delivery were higher in primiparous females (Table I). These results suggested that there may be small differences in maternal physique between primiparous and multiparous females that may be correlated with pregnancy and delivery. The maternal body accumulates fat during the second trimester, and maternal lipid metabolism then switches to a catabolic condition during the third trimester to adapt to the increased nutritional requirements of late pregnancy (25). Obesity increases the risk of diseases such as hypertension, type 2 diabetes, hyperlipidemia and arteriosclerosis (26–28). Therefore, from the perspective of disease prevention and health maintenance, it is necessary to not only control weight during pregnancy but also following delivery.

In conclusion, the results of the present study have revealed significant differences between primiparous and multiparous females, with regard to gestational weight gain and weight reduction following delivery. In addition, the study suggested that the factors influencing birth weight differed between primiparous and multiparous females. The degree of gestational weight gain and the prepregnancy physique have significant impacts on the outcome of pregnancy for the mother and the infant. Since these outcomes also affect the long-term health of the mother and child, there is a requirement for health guidance to be tailored to the individual pregnant female. To improve maternal and fetal outcomes, further studies regarding the correlations between prepregnancy physique and the degree of gestational weight gain are necessary.

## Figures and Tables

**Table I.** Summary and comparison of maternal factors in parity.

Factors	Total population (n=560)	Primiparous (n=220)	Multiparous (n=340)
Age (years)^a^	26.6±4.6	24.1±3.8	28.2±4.3^g^
Smokers^b^	108 (19.3)	51 (23.2)	57 (16.8)
Number of cigarettes/day	10.1±5.9	10.6±6.8	9.7±5.0
Prepregnancy weight (kg)^a^	53.7±9.0	52.8±8.8	54.3±9.0^e^
Height (cm)^a^	158.8±5.5	159.1±5.2	158.6±5.7
Prepregnancy BMI^a^^c^ (kg/m^2^)	21.3±3.3	20.8±3.1	21.6±3.5^f^
BMI <18.5^b^	85 (15.2)	39 (17.7)	46 (13.5)
BMI 18.5–25^b^	405 (72.3)	160 (72.7)	245 (72.1)
BMI 25 to <30^b^	53 (9.5)	17 (7.7)	36 (10.6)
BMI ≥30^b^	17 (3.0)	4 (1.8)	13 (3.8)
Delivery weight (kg)^a^	65.7±3.0	65.5±9.1	65.8±8.7
Gestational weight gain (kg)^a^	11.9±4.3	12.7±4.3	11.4±4.3^f^
Duration of pregnancy (weeks)^a^	39.5±1.2	39.5±1.2	39.5±1.2
1 month postpartum weight (kg)^a^^d^	58.4±8.2	58.1±8.5	58.6±8.1
Postpartum weight loss (kg)^a^^d^	7.3±2.3	7.6±2.3	7.1±2.3^e^

Data are presented as the mean ± standard deviation, except where stated.

a Compared using a two sample t-test;

b Presented as a number and percentage and analyzed using a χ^2^ test;

c Body mass index (BMI): Weight in kilograms divided by the square of the height in meters (kg/m^2^);

d n=491;

e P<0.05,

f P<0.01 and

g P<0.001 compared with primiparous females.

**Table II.** Summary and comparison of the infants in parity.

Factors	Total population (n=560)	Primiparous (n=220)	Multiparous (n=340)
Male^a^	285 (50.9)	115 (52.3)	170 (50.0)
Female^a^	275 (49.1)	105 (47.7)	170 (50.0)
Birth weight (g)^b^	3219.3±371.5	3153.0±364.1	3262.3±370.4^d^
Birth height (cm)^b^	49.7±1.8	49.5±1.9	49.8±1.8
Head circumference (cm)^b^	33.3±1.5	33.1±1.6	33.5±1.3^d^
Chest circumference (cm)^b^	31.7±1.7	31.3±1.8	31.9±1.6^e^
Low birth weight^a^	8 (1.4)	5 (2.3)	3 (0.9)^c^
Placental weight (g)^b^	518.0±85.2	511.9±78.7	522.0±89.0

Data are presented as the mean ± standard deviation, except where stated.

a Presented as a number and a percentage and analyzed using a χ^2^ test;

b Compared using a two sample t-test;

c P<0.05,

d P<0.01 and

e P<0.001 compared with primiparous females.
